# Vacancy Structures and Melting Behavior in Rock-Salt GeSbTe

**DOI:** 10.1038/srep25453

**Published:** 2016-05-03

**Authors:** Bin Zhang, Xue-Peng Wang, Zhen-Ju Shen, Xian-Bin Li, Chuan-Shou Wang, Yong-Jin Chen, Ji-Xue Li, Jin-Xing Zhang, Ze Zhang, Sheng-Bai Zhang, Xiao-Dong Han

**Affiliations:** 1 Beijing Key Laboratory and Institute of Microstructure and Property of Advanced Materials, Beijing University of Technology, Beijing 100124, China; 2 State Key Laboratory on Integrated Optoelectronics, College of Electronic Science and Engineering, Jilin University, Changchun 130012, China; 3 Center of Electron Microscopy and State Key Laboratory of Silicon Materials, Department of Materials Science and Engineering, Zhejiang University, Hangzhou 310027, China; 4 Department of Physics, Beijing Normal University, Beijing 100875, China; 5 Department of Physics, Applied Physics, and Astronomy, Rensselaer Polytechnic Institute, Troy, New York 12180, USA

## Abstract

Ge-Sb-Te alloys have been widely used in optical/electrical memory storage. Because of the extremely fast crystalline-amorphous transition, they are also expected to play a vital role in next generation nonvolatile microelectronic memory devices. However, the distribution and structural properties of vacancies have been one of the key issues in determining the speed of melting (or amorphization), phase-stability, and heat-dissipation of rock-salt GeSbTe, which is crucial for its technological breakthrough in memory devices. Using spherical aberration-aberration corrected scanning transmission electron microscopy and atomic scale energy-dispersive X-ray mapping, we observe a new rock-salt structure with high-degree vacancy ordering (or layered-like ordering) at an elevated temperature, which is a result of phase transition from the rock-salt phase with randomly distributed vacancies. First-principles calculations reveal that the phase transition is an energetically favored process. Moreover, molecular dynamics studies suggest that the melting of the cubic rock-salt phases is initiated at the vacancies, which propagate to nearby regions. The observation of multi-rock-salt phases suggests another route for multi-level data storage using GeSbTe.

The important technological material GeSbTe (GST), especially on the GeTe-Sb_2_Te_3_ pseudo-binary line, shows an extraordinary potential in optical/electrical applications due to the outstanding switching behavior^1^,^2^,^3^,^4^. GST is supposed to be a good candidate for the non-volatile phase change memory (PCM)^2^,^3^,^4^,^5^, and in recent years, GST have also been applied to the brain-like computing systems^6^, and electronic displays^7^. With assistance of electrical (or laser) pulse, GST can switch between the amorphous and crystalline phases within tens of nanosenconds^1^,^2^,^3^,^4^, and are accompanied by considerable changes in the resistivity or reflectivity^1^,^2^,^3^. Knowledge about the atomic structure is crucial to understand the mechanism of the fast reversible phase transformation^8^,^9^,^10^,^11^ and to further improve the performance of its relevant devices. According to the previous literatures^1^,^12^,^13^,^14^, GST possesses at least two crystalline phases, i.e., the cubic metastable and hexagonal (or trigonal) stable phases. The metastable phase has a rock-salt-like structure where anion sites are occupied by Te, and cation sites are randomly occupied by Ge/Sb plus a certain amount of vacancies^15^,^16^,^17^. The concentration of the vacancies in the cation sites depends on the chemical composition^17^, and is 20% for Ge_2_Sb_2_Te_5_. However, there is controversies with respect to the vacancy distribution, which was reported as a random distribution based on the X-ray diffraction (XRD) data^15^,^16^, but some theoretical studies predicted that the vacancies could be highly ordered and even form layers on the (111) planes^8^,^14^ or be more complicated^18^. Recently, it was proposed that vacancy ordering in cubic GST may be controlled by thermal annealing^3^,^19^ or electronic beam radiation^20^, and a vacancy semi-ordered^21^ cubic GST has been observed. The vacancy distribution affects not only the structural stability and the phase transition behavior^8^,^14^, but also the physical properties, such as the electronic^3^ and thermal^19^ transports. Another issue is the structural transition from the cubic phase to the hexagonal phase, as which share considerable structural similarities^8^,^13^, except that the vacancies are mostly ordered in the latter case.

Many efforts^9^,^10^,^11^,^22^,^23^ have been made to understand the transition between the cubic and amorphous phases of GST, in which the vacancies are believed to play a vital role^9^,^10^,^11^,^24^. In particular, amorphization (reset operation in PCM) is a key step for PCM devices, which is usually achieved through the melt-quench process by applying an intense laser (or electric) pulse^2^,^4^. Thus, the melting behavior of the GST is important for its PCM applications. In the past, studies were mainly interested in understanding the structure evolution during the melting (or disorder) of GST^25^,^26^,^27^,^28^,^29^. For example, through first-principles calculations, Sun *et al.*^25^, found a unique melting behavior of some hypothetical vacancy-ordered GST cubic structure in which some ordered structural motifs remain intact after the melting. However, the roles of the vacancies and their distribution during the real-time melting process have not been reported. Often, amorphization (or melting) can be a multi-level process^30^,^31^, which could be beneficial for GST-based PCM devices. Recently, eight-level storage and three-bits in a single cell have been reported^32^. Currently, multi-level storage^32^,^33^,^34^ has been realized by controlling the fraction of the crystalline^33^ or amorphous^35^ regions within one storage cell. However, realizing multi structures in GST could offer another way to achieve multi-level storage, for example, by creating and manipulating multi-levels in cubic GST with different degrees of vacancy ordering and resistivity^3^.

In this work, we report a combined experimental and theoretical study on the vacancy ordering in cubic GST, in connection with its role in the cubic-to-hexagonal phase transition and amorphization, and the possibility of multi-level cubic and amorphous GST phases for PCM. Using spherical aberration-corrected (Cs-corrected) high angle annular dark field scanning transmission electron microscopy (HAADF-STEM) combined with atomic energy-dispersive X-ray (EDX) mapping, we verify two states for cubic GST, one with vacancies randomly distributed (the primarily cubic phase) and one with highly ordered vacancies [the vacancy-ordered cubic (VOC) phase]. First-principles molecular dynamics (MD) studies reveal that the vacancy ordering is in fact energetically favorable. In addition, melting (or amorphization) in the cubic phases occurs at the vacancies and then propagates into nearby regions.

## Results and Discussion

**Figure 1:**
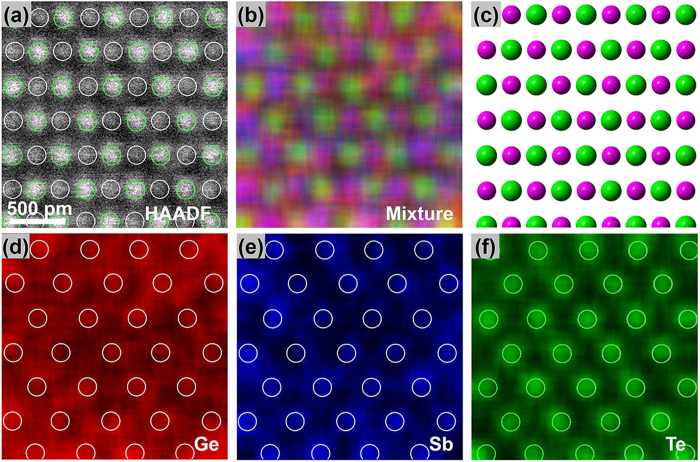
Structural and chemical identification of the GST primarily cubic structure projected along the [110] direction. (**a**) HAADF image. (**b**) The mixture EDX mapping and (**d**–**f**) the individual mappings for the corresponding element in (**a**,**c**). The corresponding schematic of atomic model with Te (green) and Ge/Sb/Vacacny (purple). The sites of Te and Ge/Sb/Vacancy are marked by green and white circles in (**a**,**d**–**f**).

Crystallization of the pulse-laser deposited (PLD) amorphous GST during *in-situ* annealing was studied by transmission electron microscopy (TEM) [see Fig. S1, Supplementary Information (SI)]. The crystallization temperature is determined to be ~150 °C, which is similar to that of the as-deposited films^1^, although the grain sizes are slightly larger ranging from dozens to hundreds of nanometers. Figure 1 shows the structure and atomic arrangement of the primarily cubic GST (160 °C -annealed), that was obtained by Cs-HAADF-STEM and EDX mapping. The HAADF image (the so-called Z contrast image) is chemically selective^36^, because the brightness of the HAADF image is roughly proportional to the square of the atomic number (Z^2^). Therefore, the appearance of the regular alternative bright (green circles) and dark (white circles) spots in Fig. 1(a) corresponds to Te and Ge/Sb/Vacancy atoms in different sites or columns (vertical to the shown plane). However, complexity due to the coexistence of Te (52), Sb (51), Ge (32) and Vacancy (0) may arise. Therefore, the atomic EDX mappings, depicted in Fig. 1(b,d–f), are considered to be more accurate and straightforward for such structural studies. These mappings confirm that Te and Ge/Sb/Vacancy atoms reside on different sublattices as reported by the previous XRD measurements^15^,^16^.

### Multi-levels in the structures in the GeSbTe cubic phase

**Figure 2:**
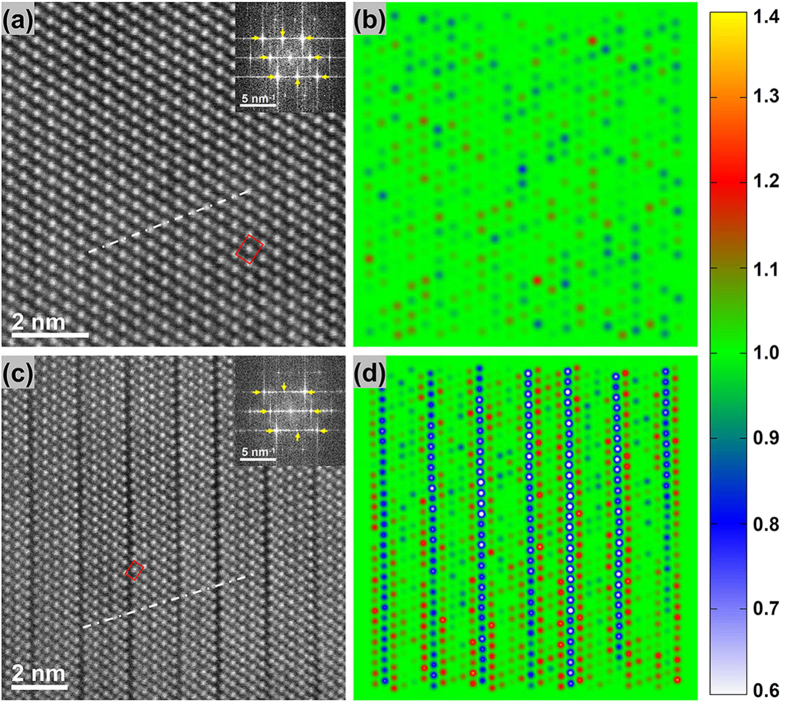
The multi-states of GST cubic phase. (**a**) The primarily cubic and (**c**) vacancy ordered cubic (VOC) phase. Their cubic structural features are highlighted and demonstrated by the white dash-dot lines and the red rectangles. The corresponding FFT patterns are shown in their insets. (**b**,**d**) are the corresponding normalized intensity mapping for Ge/Sb/Vacancy sites (to reflect the concentration of vacancy) in (**a**,**c**) with color bar on the right-side.

To investigate the multi-levels in the cubic-GST with different vacancy ordering, two samples were annealed at 160 °C and 300 °C for 10 minutes. Figure 2 shows their comparison based on the projected structures along the [110] direction. The sample annealed at 160 °C shows a homogenous structure as the primarily cubic phase [Fig. 2(a)]. However, the sample annealed at 300 °C exhibits a lamella-like structure (which is similar to the hexagonal phase), as the VOC phase [see Fig. 2(c)], where the periodically dark stripes originate from the vacancy accumulation. Before determining this VOC structure (i.e., it is the cubic or hexagonal phase), a detailed structure comparison between these two phases was performed as shown in Fig. S2 (in SI). In the cubic phase, the Te (or Ge/Sb/Vacancy) sites are always arranged in lines and in the local area five neighboring Te (or Ge/Sb/Vacancy) sites can form a rectangle with four dots as corners and one as center, see the dotted lines and rectangles in Figs 2(a) and S2(a). However, both of these structural features have been interrupted across the Te-Te vacancy layers in the hexagonal phase, see Fig. S2(b). Therefore, the two distinguishable features in the VOC phase [in Fig. 2(c)] suggest its cubic structural stacking. Namely, the VOC phase is a lamella-like cubic phase. In addition, the fast Fourier transition (FFT) patterns [in the inset of Fig. 2 (a,c)] also confirm the cubic structural similarity (as indicated by yellow arrows) between the VOC phase and the primarily cubic phase. Compared to the case in the primarily cubic phase, some extra diffraction spots (with a shorter reciprocal lattice distance compared to the original reciprocal lattice constant along the [111] direction) in the VOC phase [inset in Fig. 2(c)] correspond to the vacancy-ordered layers.

To quantitatively estimate the vacancy distribution from the HAADF images, a method based on image processing (see the details in Fig. S3 in SI) was introduced. We only picked out the Ge/Sb/Vacancy columns (sites), as shown in Fig. 2(b,d). The normalized intensity with different colors roughly reflects the relative concentration of vacancy in each Ge/Sb/Vacancy columns. Blue-to-white represents a vacancy-rich (or atom-poor) distribution. Red-to-yellow indicates a vacancy-poor (or atom-rich) distribution, and green is for an average vacancy distribution. Figure 2(b) shows a distribution with small deviation from green indicating a random distribution of vacancies and Ge/Sb atoms in the primarily cubic phase. However, Fig. 2(d) shows a series of blue-to-white columns suggesting vacancy ordering in the (111) layers in the VOC phase. Such vacancy layers divide the cubic matrix into pieces of lamellas. Interestingly, two vacancy-poor layers (red-to-yellow) are always found adjacent to the vacancy-ordering layer. In other words, vacancies in these neighboring layers tend to fully diffuse into the vacancy-ordering layer, whereas the vacancies in the far-away cation layers are more difficult to diffuse. According to the composition of Ge_2_Sb_2_Te_5_, the average concentration of vacancies in the cation sublattice is 20%^15^,^16^,^17^. As such, 60% is the expected concentration of vacancies in these (111) vacancy-ordering layers, based on the vacancies from the two adjacent layers. Of course, we cannot exclude some Sb may aggregate into the two “red-to-yellow” layers due to the large atomic number.

### Stability and distortion of the VOC phase

**Figure 3:**
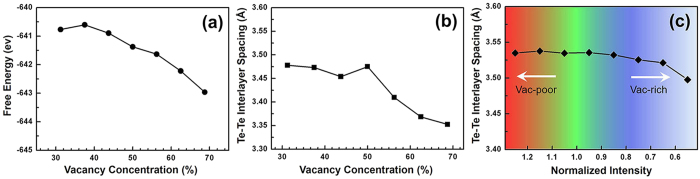
The effect of the vacancy ordering in GST cubic phases on the stability and the lattice distortion. (**a**) The calculated energy and (**b**) the Te-Te interlayer distance upon the vacancy-ordering degree on the (111) layer. (**c**) The experimentally measured Te-Te interlayer distances in the VOC phase [in Fig. 2(c)] rely on the normalized intensity mapping of Ge/Sb/Vacancy columns as those in Fig. 2(d).

In this section, we further investigated the stability and the lattice distortion of the VOC phase. The theoretical results are obtained by first-principles calculations. According to the estimation of the concentration of vacancy (~60%) in the vacancy-ordering layer, a range from 30% to 70% is used in the calculations. Figure 3(a) shows that the vacancy aggregation is energetically favored because the energy decreases with increasing concentration, which is consistent with a previous report^37^. For lattice distortion, Fig. 3(b) shows the distance between the two Te layers, which sandwich the vacancy-ordering layer, decreases with the vacancy accumulation. To estimate the lattice distortion of the VOC phase from the HAADF images, we measured the Te-Te interlayer distance and depicted in Fig. 3(c) according to the relevant vacancy concentration mapping [Fig. 2(d)]. The local Te-Te interlayer distance is indeed slightly decreased with the increase of the vacancy accumulation (the decrease of the normalized intensity is a rough indication of vacancy accumulation). It should be stressed that the Te-Te interlayer distances in the VOC phase is close to those in the primarily cubic phase but are much larger than in the hexagonal phase, as shown in Fig. S1(b).

The cubic-to-hexagonal phase transition is another key issue for GST which has been intensely studied^8^,^14^,^38^,^39^. According to the above results, we can propose a model of this phase transition. Firstly, vacancies are randomly distributed in the primarily cubic phase. Then, with annealing at high temperature (for example at 200 °C^21^), the vacancies are gradually accumulated and ordered into the (111) planes in the form of a local “vacancy plate” as a vacancy semi-ordered cubic structure^21^. With further increasing of the temperature, more vacancies are moved into the “vacancy plates”. During the vacancy ordering process, the energy is decreased and the lattice is compressed slightly along the [111] direction. When most of the vacancies are ordered in the (111) layers (for example 60%), the VOC phase is formed with lamella structures similar to those in the hexagonal phase. Since the hexagonal phase in GST has a definite number of layers in each lamella for a specific composition, such as nine layers for Ge_2_Sb_2_Te_5_ and seven layers for Ge_1_Sb_2_Te_4_. Therefore, most of the nine-layer lamellas in the VOC phase as shown in Fig. 2(c), demonstrate that the samples are indeed the Ge_2_Sb_2_Te_5_. The hexagonal phase of GST, is in the form of stacking lamellas separated by Te-Te gaps, and is always energetically favorable compared to the cubic phase. As the concentration of vacancies approaches to 100% in the layer (as a true Te-Te gap) or a critical high value, the cubic-to-hexagonal phase transition occurs by sliding the building blocks^8^ (lamellas) and collapsing the vacancy layer. Thus, the vacancy-ordering process plays a critical role in the phase switching^13^,^37^. We expect the *in-situ* observation of vacancy ordering and the phase transition to be important in future research. Currently, there are at least three cubic phases in GST: the primarily cubic phase (random distribution of vacancies), the vacancy semi-ordered^21^ cubic phase, and the VOC phase. These multi-level structures in GST hint the possible application to multi-level storage. Additionally, the VOC phase should be an important intermediate state during the cubic-to-hexagonal transition because it is compatible with the structural characteristics of both structures.

### Melting in the VOC phase

**Figure 4:**
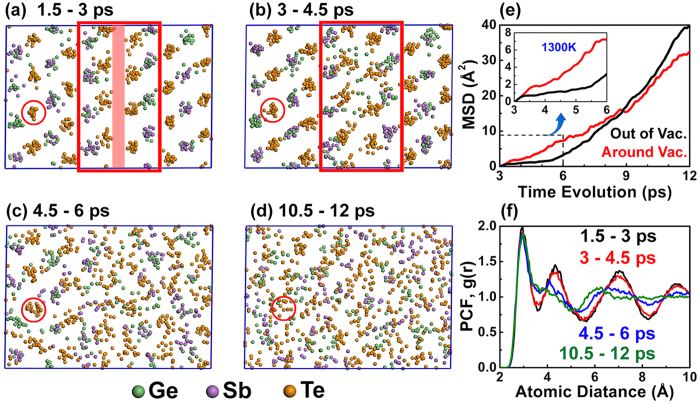
The simulation of melting process in the VOC phase. (**a**–**d**) The structural evolution during the melting process at 1300 K. Five snapshots in 1.5 ps are collected in each figure. The initial vacancy layer is highlighted by the red shading rectangle and the nearby atoms are marked by the red empty rectangle. A Te far from the initial vacancy layer is marked by a red circle. (**e**) The MSD for the atoms around the vacancy layer (inside the red empty rectangle) and out of the vacancy layer during melting process. (**f**) The PCFs with time evolution.

In PCM devices, amorphization (reset operation) is usually achieved via a melt-quenching process by an intense laser (or electrical) pulse^2^,^4^ although some reports have proposed the solid-state amorphization can also be achieved by photoassist^40^ or electronic excitation^41^. In the following section, the melting behavior of the VOC phase is investigated by first-principles MD simulation. As shown in Fig. 4(a), a supercell with 172 atoms (Ge_38_Sb_38_Te_96_) is used to mimic the VOC phase where the vacancy layer is highlighted by a red shading rectangle in Fig. 4(a). We firstly increase the temperature form 300 K to 900 K within 6 ps. Then, the annealing is performed at 1300 K for 12 ps to observe the melting process. To observe the atomic diffusion in the melt, an image at every 1.5 ps (including 5 snapshots, one per 0.3 ps) is extracted during the annealing. In the first stage [1.5–3 ps, Fig. 4(a)], all of the atoms shake intensely but the whole structure is still highly ordered, which is also reflected by its pair correlation function (PCF) in Fig. 4(f). From 3 to 4.5 ps, the atoms near the vacancy layer start to diffuse into the layer and melt, whereas the atoms far away maintain order, [Fig. 4(b)]. Therefore, the PCF maintains crystalline features. In the next 1.5 ps, the atoms far from the original vacancy layer start to diffuse significantly, and the structure becomes disordered. However, some of the atoms (far from the vacancy layer) are still ordered, such as the Te marked with a circle [Fig. 4(c)]. At 12 ps, the structure becomes completely disordered. The crystal characteristic in PCF at r >6 Å, which correspond to the long-range order, disappears [Fig. 4(f)]. The mean square displacement (MSD) during the melting process provides valuable information. We divide the MSD into two parts: one is for atoms around the vacancy layer [as shown in the red empty rectangle in Fig. 4(a,b)] and the other is for the atoms outside of the vacancy layer. Figure 4(e) shows the MSD around the vacancy layer increases more significantly before 6 ps. Therefore, the VOC phase starts to melt from the region near the vacancy layer. The MSD outside of the vacancy layer starts to increase after more time passes (i.e., >6 ps), with an indication that the melt extends to this region. This phenomenon is possibly due to the vacancy layers, which provide space for atomic diffusion.

To understand the roles of the multi-level vacancy distributions in the melting behavior in GST, we also performed the melting MD on the other two cubic models: the random vacancy distribution and the VOC phase with less ordered vacancies in the vacancy-ordering layer. As expected, Fig. S4 (in SI) shows the melting always occurs in the region near the vacancies. Compared to the case in the VOC phase, Fig. S4 shows that these two models (with less ordered distribution of the vacancies) melt faster. In other words, the VOC phase requires a longer time to become completely disordered. Thus, it may provide an opportunity to controlling the disorder degree by a series of hierarchic amorphization processes. Here, we quenched the 1300 K-annealing VOC phase at different periods (with different melting times) to 300 K. The imaginary part of the dielectric constant (ε_2_) for these quenched structures is calculated. Figure S5 (in SI) shows that ε_2_ decreases continuously with the melting time. In this way, we can obtain structures with different optical/electrical properties, which is a potential technique to achieve multi-level storage.

In summary, the multi-structural properties of cubic GST have been studied both experimentally and theoretically. In addition to the well-known cubic phase with primarily random vacancy distribution, a VOC phase is identified at elevated annealing temperature such as 300 °C. This new phase is energetically more stable but its lattice is only slightly distorted from that of the rock-salt structure. We suggest that this VOC phase, as an intermediate state between the primarily cubic phase and hexagonal phase, plays a vital role in the transition among the cubic phase, the hexagonal phase, and even the amorphous phase. Moreover, MD simulation reveals that the amorphization of the VOC phase occurs from ordered vacancies and then propagates to other regions. These findings address some of the key questions about the structural properties and amorphization behavior of the popular GST memory alloys, which could be vital for further optimization of the materials for practical PCM devices.

## Methods

### Sample preparation

The amorphous PLD-Ge_2_Sb_2_Te_5_ films with thickness of few micrometers were deposited on the Si substrate (covered with an amorphous SiO_2_ film) using a stoichiometric Ge_2_Sb_2_Te_5_ alloy target. The composition of the films were confirmed as nearly Ge_2_Sb_2_Te_5_ by energy dispersive X-ray (EDX) Spectrum. The cross sectional samples for TEM and STEM study were performed by the focused ion beam system (FIB). To minimize the beam damage during the FIB sample preparation, the multiple processes with reducing voltages and beam current are used. The parameters were 5 kV and 23 pA when the sample was thinner than 300 nm, and were further reduced to 2 kV and 16 pA as the thickness of the sample was under ~100 nm.

### TEM measurements

The *in-situ* annealing study was performed on the JEOL 2010 TEM with heating rate of 10 K/min, and hold for 10 minutes at 150 °C and 300 °C, respectively. The samples for the HAADF investigation were annealed at 160 °C and 300 °C for 10 minutes in TEM. The HAADF images and EDX mappings were obtained on a FEI Titan ChemiSTEM 80–200 Cs-TEM with a super-X detector. Both of the TEM and STEM were performed at 200 kV. The details of the image processing was described in SI in Fig. S3.

### Calculation detail

Our calculation employs density functional theory (DFT)^42^ as implemented in the Vienna Ab-initio Simulation Package (VASP) code^43^. The projector augmented wave (PAW)^44^ pseudopotential is used to describe the electron-ion interaction, and the electronic exchange-correlation interaction is described by generalized gradient approximation (GGA) with the Perdew-Burke-Ernzerhof (PBE) functional^45^. In the MD simulation, we use the NVT canonical ensemble, in which the Nosé thermostat is used to control the temperature^46^. For both structure optimize and MD simulation, we employs an energy cutoff of 200 eV, K-point set of 1 × 1 × 1, Gaussian smearing with 0.1 eV, and no spin.

### Modeling process

Our GST model is build based on the rock-salt structure. In order to form a vacancy layer penetrating the (111) plane, we redefine a new lattice vectors in the calculation cell: (1 1 1), (−1 1 0), (−1 −1 2), in terms of the rock-salt lattice. The initial lattice parameter is set to match the density measured by experiment^47^. Therefore, the parameters of initial supercell are a = 20.8369 Å, b = 17.0124 Å, c = 14.7331 Å and the model contains 38 Ge, 38 Sb, 96 Te atoms and 20 vacancies. Then the models with different degree of vacancies accumulation are optimized with volume variation.

## Additional Information

**How to cite this article**: Zhang, B. *et al.* Vacancy Structures and Melting Behavior in Rock-Salt GeSbTe. *Sci. Rep.* **6**, 25453; doi: 10.1038/srep25453 (2016).

## Supplementary Material

Supplementary Information
