# Supplementary material for: Vacancy Structures and Melting Behavior in Rock-Salt GeSbTe
Source: Sci Rep. 2016 May 3;6:25453. doi: 10.1038/srep25453 (PMC4853729; doi:10.1038/srep25453)
Supplement: Supplementary Information [file srep25453-s1.doc]

**Supplementary Materials**

**Vacancy Structures and Melting Behavior in Rock-Salt GeSbTe**

Bin Zhang,^1^ Xue-Peng Wang,^2^ Zhen-Ju Shen,^3^ Xian-Bin Li,^2^ Chuan-Shou Wang,^4^ Yong-Jin Chen,^1^ Ji-Xue Li,^3^ Jin-Xing Zhang,^4^ Ze Zhang,^1,3^ Sheng-Bai Zhang,^2,5^ and Xiao-Dong Han^1^

**1. In-situ TEM analysis on the annealing process of PLD-Ge_2_Sb_2_Te_5_**


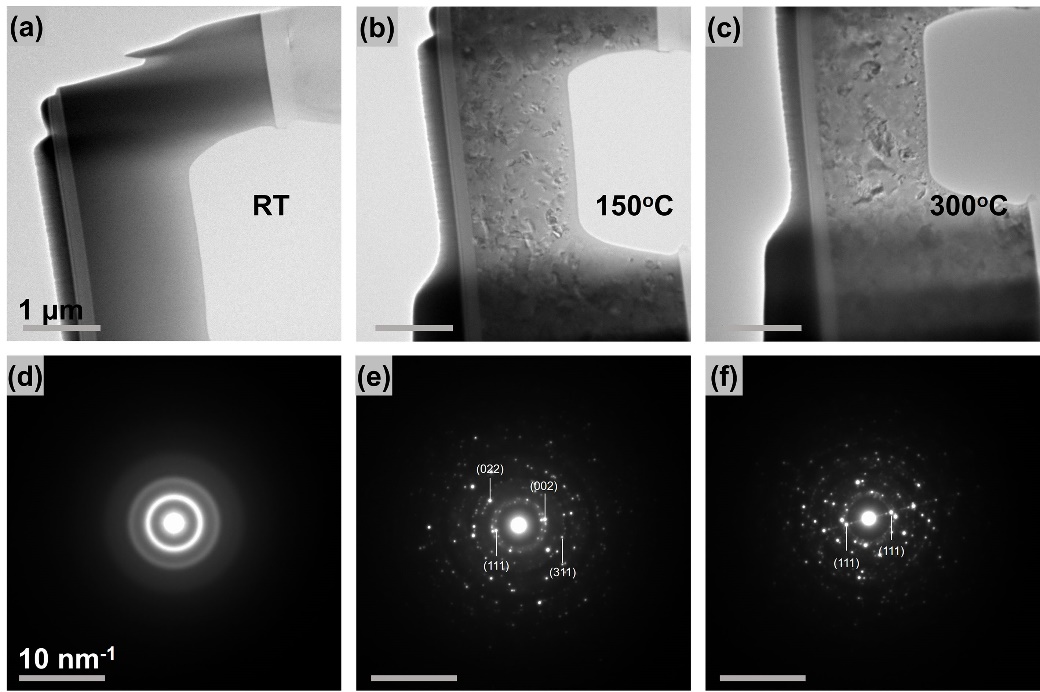


Figure S1. The in-situ annealing process of PLD-Ge_2_Sb_2_Te_5_ film by TEM analysis. (a-c) The BFTEM images and (d-f) the corresponding diffraction patterns of the GST at room temperature, annealed at 150^o^C and 300^o^C for 10mins, respectively. Bar = 1 μm for the BFTEM images and 10 nm^-1^ for the diffraction patterns.

The initial PLD-GST was confirmed as an amorphous phase with a uniform image contrast in Fig. S1(a) and the typical diffusive diffraction rings for the amorphous structure in Fig. S1(d). The crystallized temperature is determined to be ~150^o^C as it starts to crystallize [Fig. S1(b)] at this temperature, and it is consistent with that of the as-deposited films^1^. As expected, the film is crystallized to the metastable rock-salt structure, which was confirmed by the diffraction pattern [Fig. S1(e)] and the further direct HAADF observation [Fig. 2(c) in the main text]. The grain size is in a range from dozens to several hundred nanometers. Although the grain size is slightly grown with the temperature rising up, the cubic phase still not transforms to the hexagonal phase even at 300^o^C, as seen in Fig. S1(c), since the similar discontinuous poly-crystalline diffraction rings (with that at 150^o^C in Fig. S1(e)) are still kept. However, it seems a long-period-like structure is generated at 300^o^C, as the weak diffraction spots along the reciprocal [111] direction appears, seen in Fig. S1(f). This long-period-like structure is further confirmed as a kind of periodic vacancy-ordered layers in the cubic phase shown in Fig. 2(c) in the main text.

**2. Lattice comparison between the GST primarily cubic phase and the hexagonal phase.**


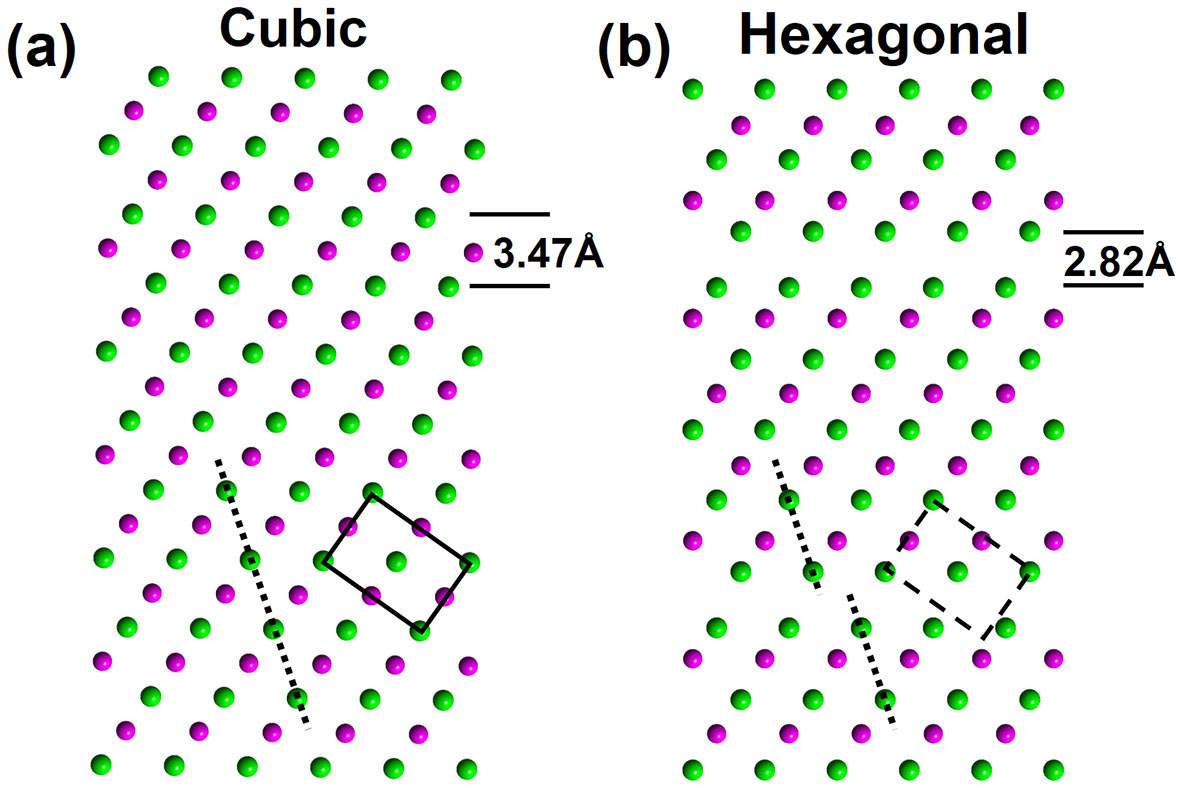


Figure S2. (a) The structure comparison between Ge_2_Sb_2_Te_5_ the primarily cubic phase (with vacancy randomly distributed) and (b) the hexagonal phase. The lattice parameters of these two models are based on the previous X-ray diffraction data^2^. The significant structural differences are highlighted by the lines and rectangles.

The projected structure of the primarily cubic phase along the [110] direction and that of the hexagonal phase along the [100] direction are shown in (a) and (b), respectively. Te is shown by the green balls while Ge/Sb/Vacancy is by the purple ones. In the cubic structure, the Te (or Ge/Sb/Vacancy) atom sites are always arranged in lines while in the hexagonal phase this in-line arrangement is interrupted by the Te-Te Van de Waals gap (of the vacancy layer). Meanwhile, in the cubic structure five neighboring Te sites could form a rectangle with four corners and one center but in the hexagonal phase it does not happen, as shown in Fig. S1(a, b). The distance of the Te-Te gaps is decreased to 2.82Å from the normal value of 3.47Å between the Te-Te layers in the cubic phase.

**3. The method for the image processing to present Ge/Sb/Vacancy columns.**


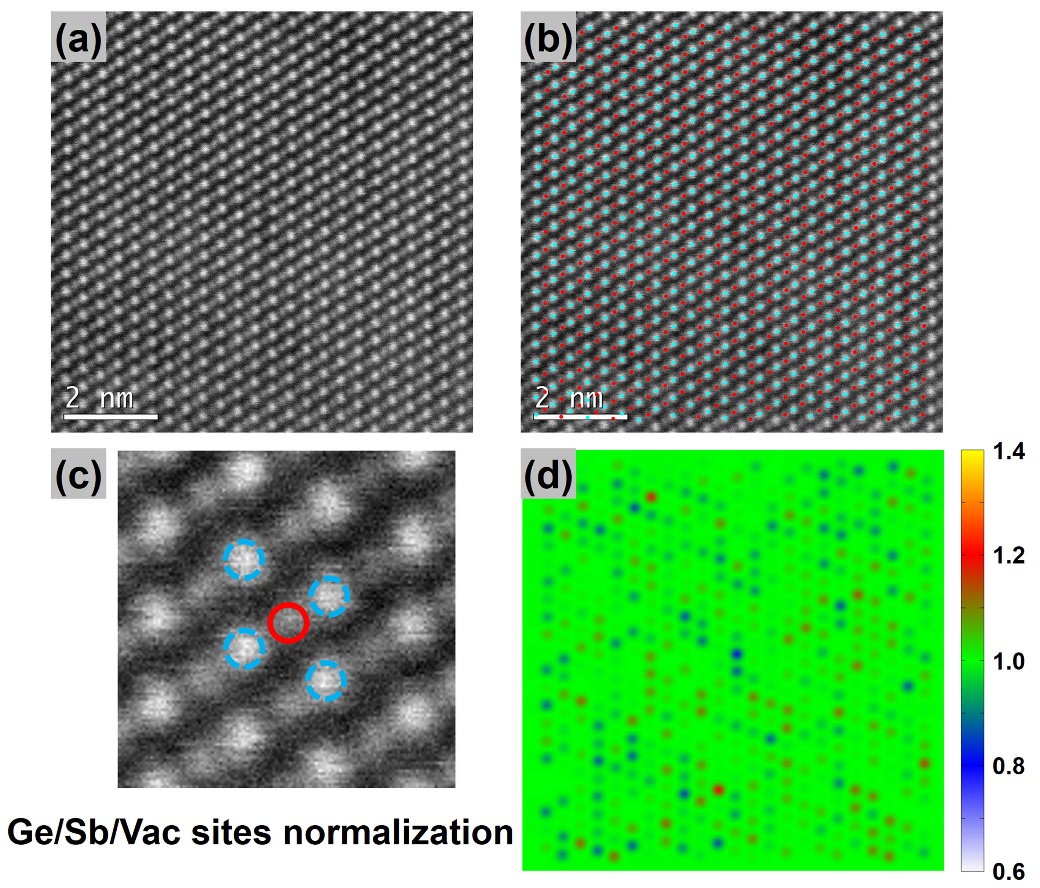


Figure S3. The image processing to show the site of Ge/Sb/Vacancy by the normalization intensity. The original HAADF image is shown in (a), and (b) the atom sites are picked out, and overlap the HAADF image. A sketch of the normalization method is shown in (c). (d) The reconstructed image where only Ge/Sb/Vacancy sites (or columns) were shown based on the normalized intensity. The color bar represents the relative brightness of Ge/Sb/Vacancy sites in the HAADF images. The average value is set to be 1.0 (green), while the value for the darker and the brighter HAADF spots vary from 0.6 (blue-white) to 1.4 (red-yellow), representing from the vacancy-rich distribution to the vacancy-poor distribution.

This image processing was used to better visualize the vacancy distribution in the HAADF images. As we known, the brightness of HAADF images is roughly proportion to the square of atomic numbers^3^. To obtain the normalized intensity of Ge/Sb/Vacancy sites, the brightness of Te is considered to be references, because they are fully occupied only by Te atoms. Generally, this image processing could be divided into three stages. First, the coordination of all atom columns is carefully picked out in Fig. S3(a) and further separated into Te (cyan dots) and Ge/Sb/Vacancy (red dots) sites, as seen in Fig. S3(b). Then, in Fig. S3(c), the intensity of Ge/Sb/Vacancy columns (the red solid circle) is divided by the average intensity of their nearest Te sites (or columns vertical to the observed plane, the four cyan dotted circles) and obtain the first-time normalized intensity of Ge/Sb/Vacancy. It’s easy to understand, the first-time normalized intensity commonly represent the relative constituent (especially the vacancy concentration) for each Ge/Sb/Vacancy sites. At last, the first-time normalized intensity is renormalized by the mean value of the whole Ge/Sb/Vacancy sites. Therefore, as shown in Fig. 3(d), the final normalized intensity is corresponding to the relative vacancy concentration in each Ge/Sb/Vacancy site or column.

**4. The melting process for the other two GST cubic structures with different vacancy-ordering degree.**


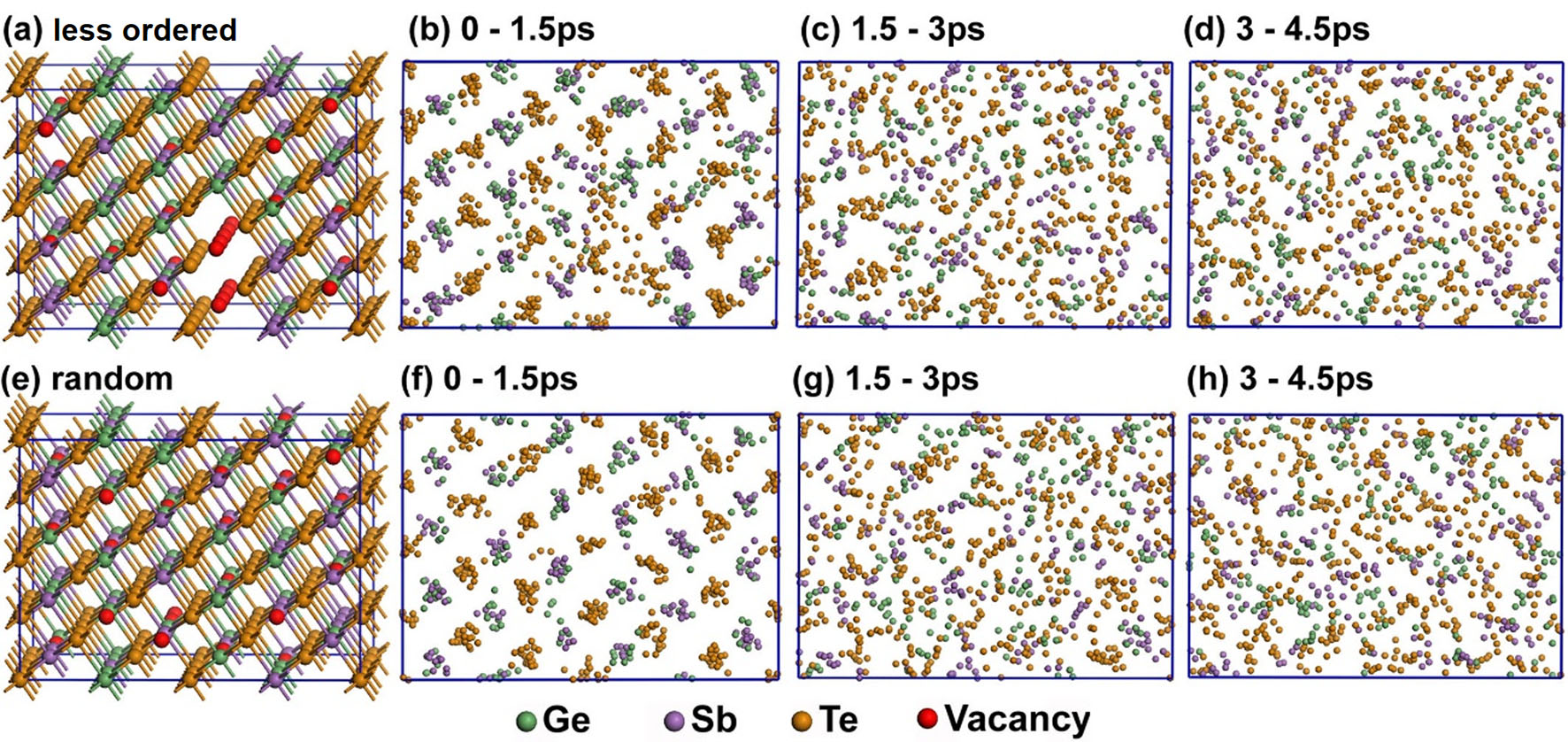


Figure S4. (a-d) The melting process for a VOC phase with less ordered vacancy distribution and (e-h) the vacancy-random cubic structure. To assist the observation, red ball is used to show the vacancies in (a) and (e).

As we have pointed out in the main text, the melting process of the GST VOC phase is started around the vacancy layer. This could be still valid for the melting process of cubic structure with less ordered vacancies. As shown in (b), the region around the vacancy firstly became disordered while the other regions are still ordered. However, both of melting in these two structures takes shorter time to become completely disordered (see (a-d) and (e-h)) than that in the VOC phase. It is believed that defects play an important role in melting process^5^. For the VOC phase, there are fewer defects in the region far from the vacancy layer. Therefore, the region far from the vacancy layer exhibits an overheating effect and takes longer time to become disordered. As a result, we can see the melting process extend from the vacancy layer to the nearby regions. When the vacancies are randomly distributed, these vacancies would act as the “liquid nucleus” and promote the melting process. That’s the reason for the cubic structures with vacancy semi-ordered and random exhibit faster melting.

**5. The Hierarchic amorphization from the different melting stage in the VOC phase and their resulting optical properties.**


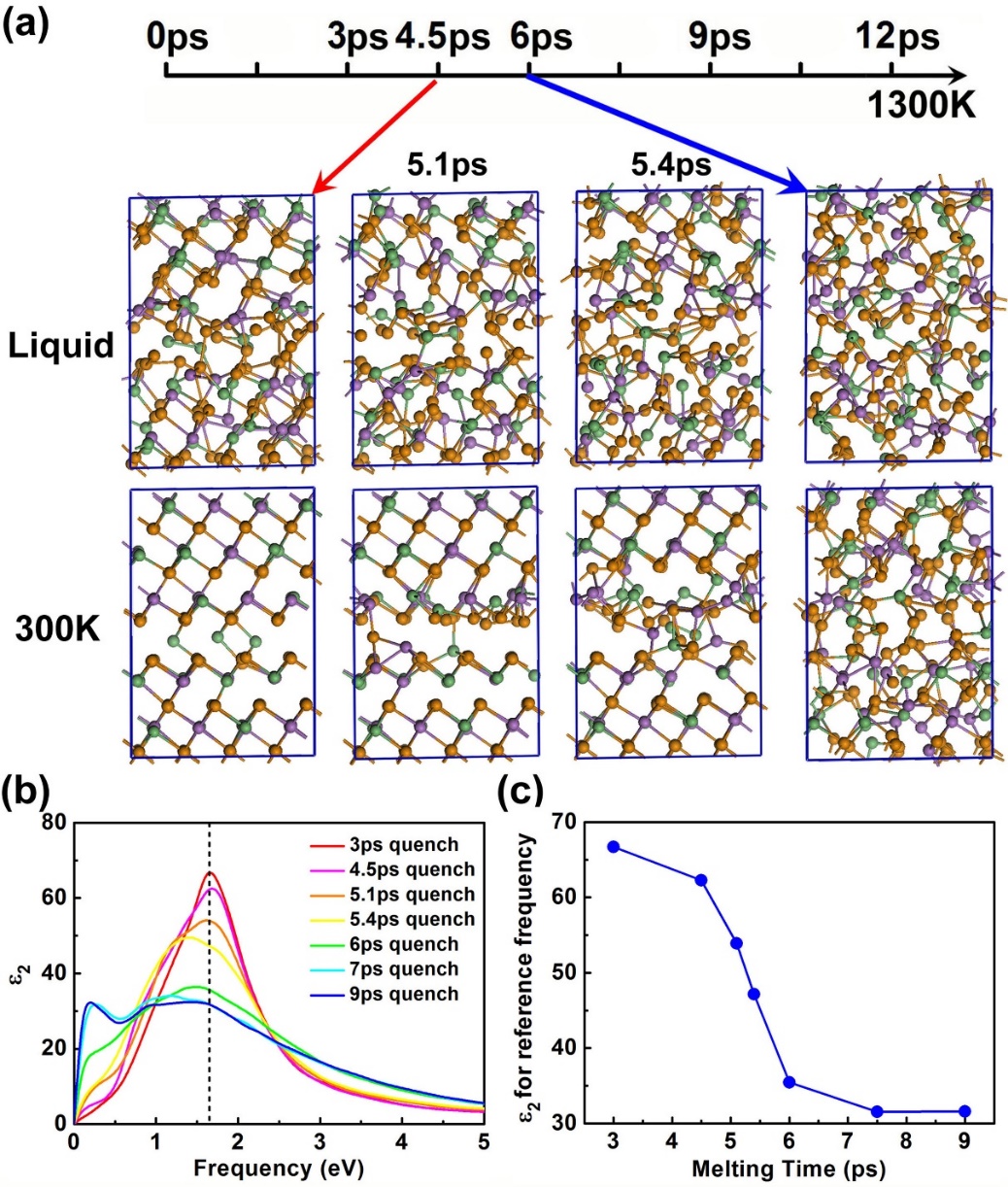


Figure S5. **Hierarchic amorphization from different melting stage in the VOC phase.** (a) Melting at different stages and the following quenched states. (b) Optical dielectric functions for the different quenched states. (c) The peaking strength in (b) varies with the melting time.

We choose the structures at different stages during the melting process at 1300K in the VOC phase. These liquid structures are rapidly quenched to 300K and maintained for 9 ps. As shown in Fig. S5(a), with a short melting time (here, less than 4.5 ps), the structure will return to the crystalline phase after the quenching, while with a longer time (more than 6 ps) the amorphous phase (with completely disordered) will obtained. We further studied the melting period between 4.5 ps and 6ps, we choose the liquid structures at 5.1 ps and 5.4 ps and quench them to 300K. In this way, we obtain two partially amorphous structures (or crystal structures with lots of defects). Because the melting process firstly takes place around the vacancy layers, the amorphous or defective region is mainly around the initial vacancy layer. Next, we calculated the imaginary part of the dielectric function (ε_2_) for each quenched structure and the results are shown in Fig. S5(b). In order to facilitate the observation, we plot the referenced peaks of ε_2_ with the melting time. In Fig. S5(c), obviously, ε_2_ shows a continuous decline with increasing the melting time. Therefore, by carefully controlling the melting process, it is possible to realize multi-level storage in the VOC phase.

**Reference**

1. Yamada, N., Ohno, E., Nishiuchi, K., Akahira, N. & Takao, M. Rapid-phase transitions of GeTe-Sb_2_Te_3_ pseudobinary amorphous thin films for an optical disk memory. J. Appl. Phys. **69,** 2849-2856 (1991).
2. Matsunaga, T., Yamada, N., & Kubota, Y. Structures of stable and metastable Ge_2_Sb_2_Te_5_ an intermetallic compound in GeTe-Sb_2_Te_3_ pseudo-binary systems. Acta. Cryst. **B60,** 685-691 (2004).
3. Penneycook, S. J. & Nellist, P. D. Scanning transition electron microscopy, Springer, (2011).
4. Yamada, N. & Matsunage, T. Structure of laser-crystallized Ge_2_Sb_2+x_Te_5_ sputtered thin films for use in optical memory. J. Appl. Phys. **88,** 7020-7028 (2000).
5. Samanta, A., Tuckerman, M.E., Yu, T.Q. & E, W. Microscopic mechanisms of equilibrium melting of a solid. Science. **346,** 729-732 (2014).
